# Causal inference and digital twins: a roadmap for the future of clinical trials

**DOI:** 10.1038/s41746-026-02871-4

**Published:** 2026-06-23

**Authors:** Silas Ruhrberg Estévez, Richard Peck, Eoin McKinney, Jim Weatherall, Stuart Bailey, Justine Rochon, Chris Anagnostopoulos, Pierre Marquet, Anthony Wood, Nicky Best, Harry Amad, Julianna Piskorz, Krzysztof Kacprzyk, Rafik Salama, Christina Gunther, Francesca Frau, Antoine Pugeat, Ramon Hernandez, Mihaela van der Schaar

**Affiliations:** 1 University of Cambridge, Cambridge, UK; 2 University of Liverpool, Liverpool, UK; 3 AstraZeneca, Cambridge, UK; 4 EMD Serono, London, UK; 5 Takeda, Cambridge, USA; 6 Quantum Black, London, UK; 7 University of Limoges, Limoges, France; 8 GSK, London, UK; 9 Accenture, London, UK; 10 Sanofi, Paris, France

**Keywords:** Business and industry, Computational biology and bioinformatics, Health care, Medical research, Scientific community

## Abstract

Clinical trials generate essential evidence on treatment safety and efficacy, but slow timelines, high costs, and limited inclusivity constrain efficiency, generalisability, and clinical impact. Causal inference and digital twins offer complementary tools to define estimands, characterise treatment-effect heterogeneity, assess transportability, and simulate patient trajectories under alternative interventions. Integrated responsibly into trial design, recruitment, monitoring, and post-trial translation, they could support faster, fairer, and more informative clinical trials.

## Introduction

Clinical trials form the foundation of modern medical practice, providing a scientifically rigorous framework for evaluating the safety and efficacy of novel treatments, drugs, and medical devices^1^. Yet they are among the most resource-intensive undertakings in healthcare innovation: late-stage (Phase III) trials frequently cost more than $500 million and require years to complete^2–4^. Inefficient trial design impacts not only budgets, but also clinician time, patient commitment, and the timely delivery of potentially life-saving therapies^5,6^. Beyond these direct costs, delayed trial completion can shorten the effective period during which new therapies can be translated into clinical use and further developed, while also postponing patient access to potentially beneficial treatments^7^.

Despite their central role in evidence generation, clinical trials often fail to capture the diversity and complexity of real-world patient populations^8,9^. More than 75% of patients are typically excluded from trial participation^10^, and as many as 30% of enrolled participants discontinue before completion^11^. Underrepresentation of key groups, including minority populations and pregnant or lactating women, remains pervasive^12,13^. While restrictive eligibility criteria are often used to reduce confounding, they can limit generalisability and slow recruitment, creating a fundamental trade-off between internal validity and real-world applicability^14^. These limitations reduce equity and may undermine safety when treatments are applied beyond trial populations^15,16^.

Recent advances in artificial intelligence (AI) have begun to transform biomedical research and clinical care^17^, from accelerating diagnosis^18^ and predicting treatment response^19^ to expediting drug discovery pipelines through in silico design^20^ and screening^21^. These developments, coupled with increasing regulatory openness to digital^22,23^ and model-informed approaches^24^, present an unprecedented opportunity to rethink how trials are designed, conducted, and analysed^25^. However, the applicability of these methods varies substantially across contexts of use, from exploratory hypothesis generation to trial design and regulatory decision-making, each of which imposes distinct requirements on data quality, validation, and interpretability.

Two AI-driven methods, causal inference and digital twins, offer promising ways to improve trial efficiency, inclusivity, and decision-making, though they have largely been applied separately in pharmacology^26–28^. Combining causal inference and digital twins enables a feedback loop between patient stratification and outcome simulation, allowing precise targeting and rapid in silico trial adaptation, an integration that is not yet systematically embedded within current Model-Informed Drug Development (MIDD) frameworks^29^. This work reflects a collaborative vision developed by leaders in pharmaceuticals, consulting, clinical research, and AI, outlining a roadmap to integrate these methods into clinical trials to support more efficient and inclusive evidence generation, while aligning with existing regulatory frameworks and contributing to the modernisation of clinical research.

### The goals of AI-enabled clinical trials

AI offers a broad spectrum of potential applications in clinical trials^25^, but this work focuses on those that accelerate development, improve success rates, and enhance data quality for fairer intervention evaluation. We distinguish three contexts of use. First, in exploratory and design-stage settings, causal inference can support biomarker discovery, enrichment, subgroup identification, and target-trial-style framing, while digital twins can enable scenario simulation, dose or regimen exploration, and protocol optimisation. Second, during trial conduct and monitoring, causal methods can help interpret emerging evidence and treatment heterogeneity, while digital twins can simulate patient trajectories under protocol-specified alternatives to inform safety monitoring and adaptive design considerations. Third, in confirmatory and regulatory settings, both methods must operate within narrow, pre-specified, rigorously validated roles. Here, causal inference is most suitable for estimand clarification, covariate adjustment, pre-specified subgroup and transportability analyses, and triangulation with trial evidence, whereas digital twins are most suitable for design support, prospective simulation, and carefully justified hybrid-control augmentation.

In addition, adaptive modifications to eligibility criteria, endpoints, or trial design in registration settings face substantial operational, ethical, statistical, and regulatory constraints, including requirements imposed by regulatory agencies and institutional review boards. While such flexibility can be valuable in exploratory and early-phase studies, its use in pivotal trials is necessarily limited and must be pre-specified, justified, and aligned with statistical and regulatory guidance. Accordingly, the most immediate opportunities for integrating causal inference and digital twins lie in design-stage optimisation, early-phase exploration, and post hoc or parallel analyses, with narrower and more tightly governed roles in confirmatory settings.

### Improving probability of success

This is the single most effective way to improve pharmaceutical R&D efficiency^30^. Causal inference can play a key role in evaluating the likelihood of true benefit rather than spurious or chance findings and better contextualise early study results^31^. Early termination of ineffective treatments improves efficiency, while causal inference and digital twins can boost success by identifying responsive subpopulations and optimal regimens for promising therapies, particularly in early-phase and exploratory settings where flexible modelling can inform downstream trial design.

### Accelerating answers

Traditional trials are slow and face challenges in evaluation, recruitment, and dose optimisation. Causal inference can pinpoint likely responders, while digital twins simulate patient trajectories to guide real-time decisions and enable adaptive trial designs, though such adaptations must be carefully pre-specified and validated to meet regulatory requirements in late-stage trials.

### Decreasing resource use

Longer trials cost more^32^, but AI can reduce expenses by accelerating timelines. Digital twins can support the development of synthetic or hybrid control arms, which may reduce sample size and recruitment requirements, particularly in exploratory settings or as augmentation to standard-of-care controls, though fully synthetic control arms remain an area of ongoing regulatory evaluation.

### Balancing confirmation and discovery

Pivotal trials often use narrow endpoints to reduce bias, but this limits exploration of broader effects. AI can expand enquiry while preserving confirmatory integrity, as causal inference reveals treatment–context interactions and digital twins enable in silico testing of alternative scenarios, supporting hypothesis generation, protocol optimisation, and better data use, while maintaining clear separation between exploratory analyses and confirmatory endpoints in regulatory settings.

**Fig. 1:**
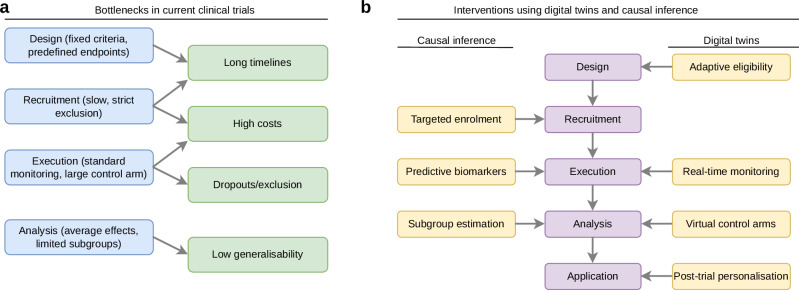
Challenges in current clinical trials and opportunities for AI-driven solutions. **a** Current clinical trials face persistent limitations in efficiency, inclusivity, and decision-making power, driven by high costs, long timelines, and underrepresentation of real-world patient populations. **b** Causal inference and digital twins can address these gaps by enabling precise patient stratification, simulating treatment outcomes, reducing reliance on large control arms, and supporting adaptive designs. Together, these methods offer a pathway to trials that are faster, fairer, and more informative.

Achieving these goals requires methodological advances, quality data, and integration into existing trial workflows. Figure 1 outlines key challenges and AI-driven solutions, while Table 1 highlights stakeholder value. Together, these methods create a powerful feedback loop: causal inference determines *who* to target by identifying subpopulations most likely to benefit, while digital twins project *how* those specific patients will respond under various counterfactual scenarios, enabling highly adaptive trial designs. Realising these benefits in practice depends on access to high-quality, appropriately governed data, as well as interoperability across clinical and real-world data sources.

**Table 1:** Potential impact of combining causal inference and digital twins across stakeholders

Stakeholder	Integrated benefit	How causal inference contributes	How digital twins contribute
Regulators	More generalisable, verifiable evidence that meets approval standards while reducing control arm size	Identifies subgroups where treatment benefit is robust across datasets	Simulates counterfactual outcomes for synthetic control arms to supplement pivotal data
Payers	Clearer definition of cost-effective patient segments	Quantifies treatment effect heterogeneity and economic impact in subpopulations	Projects long-term real-world outcomes and adherence scenarios for those segments
Clinicians	Personalised treatment sequencing and proactive safety management	Pinpoints predictive biomarkers and interaction effects	Models individual trajectories to optimise dosing and flag safety risks early
Patients	Expanded access with reduced exposure to ineffective or harmful interventions	Identifies high-benefit profiles even in underrepresented populations	Simulates safety and efficacy in complex cases to inform eligibility decisions
Funders	Faster, more efficient R&D with higher probability of late-phase success	Improves early go/no-go decisions by quantifying likely benefit	Tests alternative trial designs and regimens in silico before costly implementation

Causal inference identifies patient subgroups most likely to benefit, while digital twins simulate their responses under varied conditions. This integration creates a feedback loop that supports adaptive, efficient, and targeted decision-making aligned with the needs of regulators, payers, clinicians, patients, and funders.

### Digital twins: transforming clinical trials for patient-centred outcomes

Pharmacological digital twins are computational models that predict individual or population-level treatment responses under specified interventions. Traditionally, such models have often relied on mechanistic equations, which provide interpretability and biological grounding but may struggle to capture the full heterogeneity and complexity of real-world clinical populations^33^. Data-driven or hybrid approaches can integrate diverse sources that traditional models often overlook such as health records, genomics, and wearables, capturing variability and better addressing clinical trial challenges^34^.

### Enhancing safety and trial diversity using digital twins

Digital twins can simulate patient-specific responses to an intervention before it is administered, offering a powerful tool for participant stratification and eligibility decisions. For example, a digital twin could model the expected pharmacokinetic and pharmacodynamic profile of a treatment in an individual with multiple comorbidities, predicting potential adverse events or suboptimal responses^35^. Rather than excluding high-risk patients, trials could enrol them with tailored monitoring based on predicted risks, improving both safety and inclusivity.

By enabling personalised risk–benefit assessments, digital twins can enhance trial diversity and may even improve enrolment by highlighting individualised benefits such as optimal dosing or monitoring plans. Deep learning models extend this further by predicting responses to untested regimens, aiding decision-making in rare or complex cases^36–38^.

During a trial, ongoing simulations can act as an early warning system. In late-stage or registration trials, however, such adaptations must be pre-specified and implemented within approved protocols to preserve statistical validity and regulatory compliance. If a patient’s digital twin indicates a high likelihood of toxicity or deterioration under the current protocol, these insights could inform decisions such as dose modification, additional monitoring, or consideration of alternative treatment strategies, particularly in exploratory or adaptive trial settings. This not only improves participant safety but can also reduce attrition, preserving statistical power^39^. Additionally, even if some trial drop-outs cannot be prevented, digital twins may play a role in making full use of the data already collected from such participants^40^. If a participant withdraws, their validated twin can continue simulating their likely trajectory, enabling analysis of full trial populations without imputation or data loss. Realising these capabilities in practice depends on access to high-quality patient-level data, as well as appropriate data governance, interoperability across sources, and regulatory-compliant data use.

### Increasing the efficiency and diversity of clinical trials with digital-twin-based control arms

Randomised control arms are the gold standard for causal inference, but they pose challenges such as the need for large cohorts^11^ and participant reluctance to risk placebo or standard-of-care assignment^41^. Digital twins offer a potential solution by creating virtual control arms^42,43^. These synthetic cohorts are generated by simulating the counterfactual outcomes of participants who receive the active intervention, allowing components of the control arm to be augmented or partially replaced^44^. For example, recent work in Alzheimer’s disease has explored the use of digital twin–based approaches to improve trial efficiency, and the European Medicines Agency (EMA) has issued a qualification opinion for prognostic covariate adjustment (PROCOVA)^45^, a methodology that leverages model-based predictions from historical data. Together, these developments illustrate a concrete pathway for regulatory engagement with digital twin–adjacent methods, demonstrating how such approaches can enhance statistical power and reduce sample size while requiring clearly defined contexts of use and rigorous validation.

Digital twins have successfully retrospectively approximated trial outcomes from observational data alone^46^, demonstrating their potential to augment or partially replace control groups. For example, methods such as SyncTwin enable the reconstruction of in silico trials by estimating counterfactual outcomes from longitudinal real-world data^46^. In a large-scale study emulating the Heart Protection Study using UK electronic health records, SyncTwin recovered treatment effects on LDL cholesterol that closely matched those observed in the original randomised trial. More recently, trial-level digital twins have also been used to examine how the effects observed in randomised studies may translate to different trial and real-world populations, including in cardiovascular disease, neurodegenerative conditions such as Alzheimer’s disease, and acute myeloid leukaemia, further supporting their role in evaluating generalisability across patient groups^47^.

In addition, given this increased efficiency using partly virtual control arms, digital twins can enable trials to incorporate multiple comparator arms, enhancing the relevance and generalisability of results and minimising the cost of clinical evidence^48^. Traditional trials typically choose comparators based on regulatory requirements, which may not reflect the most clinically or regionally relevant treatments^49^. Hybrid control designs using digital twins can bridge this gap, enhancing the global applicability of findings while reducing resource demands.

Despite their promise, digital twin approaches face important limitations^50,51^. Their performance depends critically on access to high-quality, comprehensive patient-level data, and may degrade when key variables are missing, mismeasured, or inconsistently recorded across data sources. Incomplete coverage of patient populations can limit generalisability, particularly when models are applied to groups underrepresented in training data^52^. In addition, rigorous validation is required to ensure that simulated trajectories accurately reflect real-world outcomes, especially when used to inform trial design or clinical decision-making. The reliance on observational data introduces the potential for bias and model misspecification, reinforcing the importance of combining digital twins with causal inference and prospective validation where possible.

### From trials to clinical practice: extending the role of digital twins

While our focus here is on how causal inference and digital twins can improve the design and conduct of clinical trials, it is worth noting that these same methods can also support post-trial deployment. In trials, digital twins can be used to optimise treatment selection and adapt trial protocols in real time. Post-marketing, the same capabilities can be extended to simulate treatment strategies across diverse patient profiles, helping clinicians personalise regimens and integrate novel therapies into complex care pathways^42,53^.

For example, in chronic disease management, a patient’s digital twin could model long-term outcomes under alternative treatment sequences, accounting for comorbidities, adherence, and lifestyle. Simulations with digital twins can provide principled platforms for decision-support in such cases, supporting more individualised treatment decisions^54^, particularly for novel therapies where the best sequencing or combination policies are not yet established.

The greatest value, however, emerges when digital twins are informed by insights from causal inference. Subgroup definitions derived from heterogeneous treatment effect estimation can guide which patient trajectories are most relevant to simulate, ensuring that twin-based predictions address clinically meaningful and biologically plausible scenarios. In this way, we can move beyond applying population mean estimates of benefit to capturing treatment effects for individuals. In turn, simulation outputs can feed back into causal models, refining estimates of treatment benefit and informing real-time adaptation.

While such model-informed insights can support monitoring and hypothesis generation, their use to directly guide patient-level decisions within a trial must be carefully governed. In particular, acting on model outputs in a way that alters treatment allocation, dosing, or monitoring intensity may require explicit pre-specification and regulatory approval.

### Causal inference: transforming clinical trials into engines of insight

Causal inference encompasses a suite of statistical and machine learning methods designed to estimate treatment effects under explicit assumptions about confounding, selection bias, treatment assignment, missingness, and measurement^55^. Unlike traditional statistical approaches that focus primarily on associations, causal inference methods aim to answer the counterfactual question^56^: *What would have happened to this patient if they had received a different treatment?* Causal inference can be used to optimise trial design, identify predictive biomarkers, and improve the transportability of findings to broader populations^57^. When integrated with AI-driven discovery tools, such as symbolic regression and differential equation discovery, causal inference can help distinguish predictive patterns from intervention-relevant hypotheses, extending the scientific value of clinical trial data beyond primary endpoint evaluation.

### Identifying predictive biomarkers through causal inference

Machine-learning–empowered causal inference methods can analyse large, complex datasets to identify candidate predictive biomarkers that may modify treatment effects, rather than merely predict baseline risk^58,59^. For example, the Oncology Biomarker Discovery (OncoBird) framework has been applied to randomised phase III oncology trial data to identify predictive biomarkers and treatment interactions, demonstrating how causal-style subgroup analysis can reveal clinically actionable response patterns within molecularly characterised trials^60^.

By leveraging large-scale datasets that would otherwise remain untapped, such as electronic health records (EHRs), biobank repositories, or data from failed and negative trials, ML-empowered causal inference methods can systematically evaluate large numbers of candidate variables, accounting for complex patient histories and a broad range of covariates. Advanced methods, including causal forests and neural network–based representation learning, can uncover complex nonlinearities and interactions that simpler models often miss^61–63^. As a result, teams can isolate biomarkers that meaningfully modify heterogeneous treatment effects (HTE), providing a principled basis for inclusion in prospective studies^64,65^.

### Subgroup analysis for tailored treatment

Beyond biomarker identification, causal inference can characterise subgroups with distinct treatment responses based on demographic, clinical, or genomic features. Traditional analyses of RCT data typically produce a single average treatment effect (ATE), collapsing heterogeneous responses into a single, often overly generic measure. In contrast, conditional average treatment effect (CATE) estimation, facilitated by ML methods, estimates how average treatment effects vary across patient characteristics. By shifting from a single-point summary to a rich treatment-response surface, CATE estimation supports a data-driven approach to trial design that informs more individualised treatment or dosing recommendations^66,67^. Recent work has further demonstrated how causal survival forest approaches can be used to estimate treatment-effect heterogeneity in a clinical trial of aldosterone antagonists for heart failure^68^, illustrating the potential of such methods to support more tailored treatment decisions beyond average effects.

Crucially, CATE estimation need not rely solely on data from a single RCT. Integrating trial data with high-quality observational sources can refine subgroup definitions and improve generalisability. In developing a new therapy, funders could merge RCT data with real-world EHRs to move beyond identifying a single “optimal” biomarker-defined group, instead uncovering a nuanced set of patient profiles who exhibit especially pronounced treatment gains. In regulatory settings, such subgroup analyses must be pre-specified or appropriately controlled to mitigate issues of multiplicity and false discovery, particularly in late-stage trials.

### Discovery-driven insights from clinical trial data

Real-world populations often differ from trial cohorts, but ML-based causal inference can help address this gap by using large observational datasets and applying transportability analyses with robust confounding adjustment to align trial findings with broader patient groups^69,70^. Traditional physiological and pharmacological models rely on predefined structures that often fail to capture patient-level variability or novel interactions. Machine learning techniques can complement these models by revealing data-driven relationships that may not be captured by predefined models^71^.

For example, symbolic regression in an anti-inflammatory trial might reveal interactions between BMI, genetics, and metabolism, identifying candidate efficacy biomarkers. Personalised ODEs bridge traditional modelling with treatment effect estimation, capturing patient variability to enable more accurate, individualised treatment^72^. However, it is important to distinguish between exploratory subgroup discovery and subgroup claims intended to support regulatory decisions. While the former can leverage flexible machine learning approaches and diverse data sources, the latter require strict control of false discovery, pre-specification, and, in many cases, prospective validation.

Coupled with digital twins, refined subgroups can be stress-tested under alternative treatments or doses, helping assess the stability of causal insights before live trials. Simulation results can then be used to stress-test causal assumptions, explore alternative trial designs, and identify settings where prospective validation is needed. However, these approaches rely on key assumptions, including adequate adjustment for confounding and sufficient overlap between treatment groups, and may be sensitive to biases in observational data. Model misspecification, unmeasured confounding, and dataset shift can affect the reliability of estimated treatment effects, highlighting the need for careful validation and, where possible, prospective confirmation.

### A framework for robust validation of AI methods in clinical trials

**Fig. 2:**
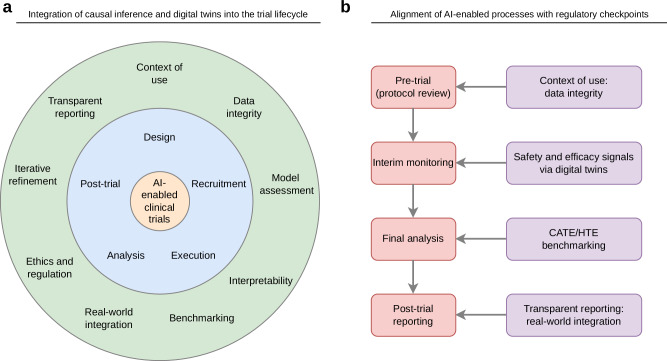
An AI-enabled framework for integrating causal inference and digital twins into the clinical trial lifecycle. **a** A structured roadmap for embedding AI methodologies into trial design, conduct, and post-trial deployment. **b** Causal inference identifies treatment–patient matches and refines subgroup definitions, while digital twins simulate personalised trajectories to inform eligibility, dosing, and safety monitoring. The framework emphasises rigorous validation, regulatory alignment, and stakeholder trust, ensuring that AI augments rather than disrupts established clinical research standards.

Successful AI integration into clinical trials requires rigorous validation to ensure reliability, generalisability, and regulatory acceptance. Figure 2 outlines how AI can augment existing workflows while preserving core principles of trial governance, statistical validity, and regulatory accountability. Validation should begin with a clearly defined context of use, specifying the model’s role and the decisions it supports.

Validation then requires careful attention to data fitness. Training and testing datasets should be representative of the target population, sufficiently complete for the intended use, and aligned with the clinical variables, outcomes, and time horizons relevant to the trial. Once data fitness has been established, models should be evaluated for predictive accuracy, calibration, robustness, and performance under missing, noisy, or atypical inputs. For causal inference methods, validation must also examine the plausibility of key assumptions, including exchangeability, positivity, consistency, measurement alignment, and robustness to unmeasured confounding or dataset shift.

Equally important is interpretability. Outputs must be understandable and actionable for clinicians, investigators, statisticians, and regulators. Benchmarking against standard statistical or pharmacological modelling approaches can demonstrate whether AI methods add value beyond existing practice. Prospective or retrospective integration studies can then test whether performance translates beyond controlled development environments into realistic trial workflows.

Ethical and regulatory concerns must also be addressed, including privacy, fairness, and algorithmic bias. Models should undergo iterative refinement, and all validation procedures should be transparently reported. Embedding this validation process within established regulatory pathways, for example, aligning with the FDA’s MIDD Pilot Programme^73^ and EMA’s Qualification of Novel Methodologies^74^, will be essential for early adoption. Demonstrating that AI-derived insights can be audited, reproduced, and explained in the same manner as conventional statistical analyses will be critical for achieving regulatory acceptance.

Realising the potential of causal inference and digital twins also requires secure, standardised data frameworks. A key practical constraint is the availability and usability of patient-level data required to parameterise and validate digital twin models. Data generated in research-use-only settings, including exploratory biomarkers or heterogeneous real-world datasets, may be valuable for model development and hypothesis generation, but may not meet the standards required for regulatory decision-making. Conversely, regulatory use typically requires well-characterised, prospectively collected, auditable datasets with clear lineage, governance, and documentation.

Data collected within a trial can often be used with appropriate consent, but extending models with external sources such as electronic health records, registries, or biobanks raises challenges related to governance, interoperability, linkage, and consent. De-identified patient-level data can support model development and validation, but may limit longitudinal linkage across sources or incorporation of time-varying covariates at the individual level. Aggregate data may be useful for benchmarking and population-level calibration, but is generally insufficient for patient-specific simulation. These constraints are particularly acute in rare disease settings, where registries and biobanks can provide valuable data but are often fragmented or de-identified. Addressing these limitations will require robust data infrastructures, privacy-preserving linkage methods, clearly defined data access frameworks, collaboration with statisticians and trial methodologists, and interdisciplinary training across AI, clinical, and regulatory domains.

### Expert recommendations for AI-enabled clinical trials

#### Pre-trial phase

Before a study begins, data scientists, biostatisticians, and clinicians could co-develop digital twin models using large, representative datasets, with retrospective benchmarking to ensure accuracy and generalisability. Causal inference experts can help define endpoints and refine eligibility via target trial emulation. Early engagement with regulators and ethics committees is essential to align on validation, transparency, and consent for data use, model development, and model-informed trial procedures.

#### In-trial phase

During trials, statisticians, monitoring committees, and modelling teams may use pre-specified digital twin outputs to support monitoring and adaptive decision rules, provided these uses are prospectively defined and statistically governed. Investigators can use these simulations for response-adaptive randomisation, arm dropping, and sample size re-estimation, while causal experts apply estimands and mediation analyses to contextualise effects and inform safety and efficacy assessments.

#### Post-trial phase

After trial completion, modelling teams, causal inference specialists, and health economists should refine digital twin and causal frameworks using the full dataset to simulate counterfactual scenarios such as alternative dosing strategies, treatment sequences, or patient subgroups not fully represented in the trial. These simulations can inform payers and health technology assessment bodies on cost-effectiveness and expected outcomes in diverse healthcare settings. The feasibility of these approaches will depend on access to high-quality data, appropriate governance frameworks, and rigorous validation consistent with regulatory expectations. Regulators should review assumptions, validations, and subgroup analyses before approving use in clinical or policy settings. Clinicians and patient groups should be involved in transparently sharing model design and validation to ensure trust and usability. This phase closes the loop between discovery and deployment, embedding AI insights into clinical guidelines and practice.

### Conclusion

Causal inference and digital twins are complementary AI-enabled methods with the potential to address long-standing challenges in clinical trials. By improving efficiency, expanding inclusivity, and enabling more precise, patient-centred insights, they offer a path to modernising evidence generation. Realising this potential will require careful integration into established trial frameworks, rigorous validation, and alignment with evolving regulatory standards. Interdisciplinary collaboration will be essential to ensure these tools enhance rather than disrupt the scientific and ethical foundations of clinical research. Early applications in high-impact areas such as oncology and rare diseases could serve as proof-of-concept, building confidence and guiding best practice. If implemented to their full potential, these approaches could contribute to establishing a new gold standard for evidence generation with faster, more equitable trials that deliver deeper insights to support truly personalised medicine.

## Data Availability

No datasets were generated or analysed during the current study.
